# A Trinuclear Oxo-Chromium(III) Complex Containing the Natural Flavonoid Primuletin: Synthesis, Characterization, and Antiradical Properties

**DOI:** 10.3390/molecules20046310

**Published:** 2015-04-10

**Authors:** Anamaria D. P. Alexiou, Carla C. Decandio, Sabrina da N. Almeida, Marcelo J. P. Ferreira, Paulete Romoff, Reginaldo C. Rocha

**Affiliations:** 1 Escola de Engenharia, Universidade Presbiteriana Mackenzie, Rua da Consolação 930, São Paulo, SP CEP 01302-000, Brazil; E-Mails: carla.decandio@ufabc.edu.br (C.C.D.); sabrina-nobrega@usp.br (S.N.A.); paulete.romoff@mackenzie.br (P.R.); 2 Departamento de Botânica, Instituto de Biociências, Universidade de São Paulo, Rua do Matão, 277, São Paulo, SP CEP 05508-090, Brazil; E-Mail: marcelopena@ib.usp.br; 3 Los Alamos National Laboratory, Los Alamos, NM 87545, USA; E-Mail: rcrocha@lanl.gov

**Keywords:** antiradical activity, chromium(III) complexes, flavonoids, metal-oxo cluster, primuletin

## Abstract

A new trinuclear oxo-centered chromium(III) complex with formula [Cr_3_O(CH_3_CO_2_)_6_(L)(H_2_O)_2_] (L = 5-hydroxyflavone, known as primuletin) was synthetized and characterized by ESI mass spectrometry, thermogravimetry, and ^1^H-NMR, UV-Vis, and FTIR spectroscopies. In agreement with the experimental results, DFT calculations indicated that the flavonoid ligand is coordinated to one of the three Cr(III) centers in an *O,O*-bidentate mode through the 5-hydroxy/4-keto groups. In a comparative study involving the uncoordinated primuletin and its corresponding complex, systematic reactions with the free radical 2,2-diphenyl-1-picrylhydrazyl (DPPH) showed that antiradical activity increases upon complexation.

## 1. Introduction

Polyphenolic compounds abound in nature and are found in fruits and vegetables, as well as in green and black tea, olive oil, wine and chocolate. The main compounds within this group are the flavonoids, which possess C_6_-C_3_-C_6_ units with each C_6_ representing an aromatic ring. Several reported studies have demonstrated that such compounds show cytoprotective and antioxidant properties [1]. Among the accepted mechanisms to explain the antioxidant activity of polyphenols is the involvement of metal coordination and scavengers of reactive oxygen species produced in living organisms [2]. The hydroxyl groups of flavonoids can bind to metal ions and, as a result, lead to an enhancement of antioxidant properties. Studies with rutin have shown that coordination with copper enhances the ability to scavenge radicals as compared to the free flavonoid [3]. The flavonoids quercetin, galangin, and catechin also have the antioxidant activity increased by coordination with iron(III), aluminum(III), zinc(II), and copper(II) [4]. Although most studies involve coordination with quercetin, other less studied flavonoids have attracted interest. For example, Li *et al*. showed that complexes formed from Ni(II), Cu(II), and Zn(II) with a naringenin Schiff base are more active in the suppression of O_2_^−^ and HO• radicals [5].

Chromium(III) is an essential metal ion involved in the metabolism of sugar and fats. Chromium-containing nutritional supplements, especially in the form of picolinate (*i.e.*, [Cr^III^(pic)_3_]), have been used since the 1980s to improve glucose metabolism, reducing fat and increasing the amount of muscle. Since the use of [Cr^III^(pic)_3_] at high concentrations can be genotoxic and mutagenic, new forms of the supplement have been proposed. These include the trinuclear chromium(III) propionate complex [Cr_3_O(EtCO_2_)_6_(H_2_O)_3_]^+^, which reduces blood levels of cholesterol and triglyceride in healthy and type II diabetic mices, and thus has been proposed as a structural and functional mimetic compound of chromodulin, a peptide containing Cr(III) [6].

**Figure 1:**
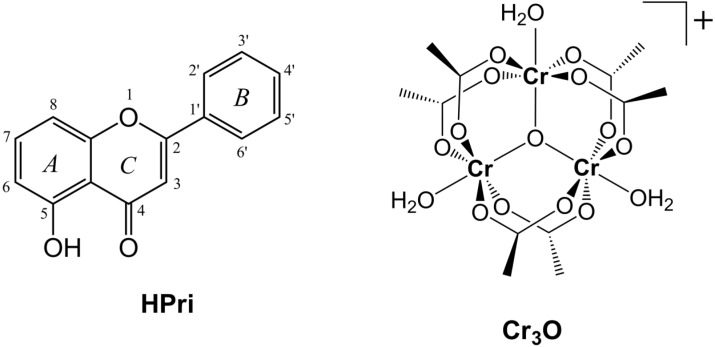
The flavonoid primuletin (HPri) and starting complex [Cr_3_O(CH_3_CO_2_)_6_(H_2_O)_3_]^+^ (Cr_3_O). Upon metal coordination, primuletin as ligand is deprotonated (Pri^−^).

In order to investigate how the coordination of a representative flavonoid with trinuclear chromium(III) complexes affects their antiradical properties, in this work we have synthesized and characterized a new complex with primuletin (5-hydroxyflavone) as the ligand (Figure 1). The product, which has a formulation [Cr_3_O(CH_3_CO_2_)_6_(Pri)(H_2_O)_2_] with primuletin (HPri) in its deprotonated monoanionic state (Pri^−^), was prepared from [Cr_3_O(CH_3_CO_2_)_6_(H_2_O)_3_]Cl [7] and structurally analyzed by the methods detailed in the Experimental Section.

## 2. Results and Discussion

The complex was found to be almost insoluble in most common solvents, with best solubility in dimethylsulfoxide (DMSO). Thus, its molar conductivity was obtained by comparison with a 10^−5^ M solution of KCl, and not with a 10^−3^ M solution as described in the literature [8]. Consistent with the molecular formulation of the neutral complex [Cr_3_O(CH_3_CO_2_)_6_(Pri)(H_2_O)_2_], the obtained low conductivity value (65 S cm^2^∙mol^−1^) indicates that this species behaves as a non-electrolyte.

The ESI mass spectrum of [Cr_3_O(CH_3_CO_2_)_6_(Pri)(H_2_O)_2_] (Figure S1) displays a base peak with *m/z* of 739.5 for [Cr_3_O(CH_3_CO_2_)_5_(Pri)(H_2_O)_2_]^+^ and several other peaks assigned to fragmented species, such as [Cr_3_O(CH_3_CO_2_)_6_]^+^ with *m/z* of 526.0. The data thus confirm that the trigonal oxo-cluster core (Cr_3_O) is maintained in the complex.

The thermogravimetric (TG) curve of primuletin showed that this compound is stable up to 190 °C, while the starting complex [Cr_3_O(CH_3_CO_2_)_6_(H_2_O)_3_]Cl presented mass loss from the beginning of heating. In the region of 25–158 °C, this precursor showed a mass loss of 14.5%, which corresponds to the release of water molecules and Cl^−^ as chlorine. The loss of acetates begins at 158 °C, with the release of four groups below 600 °C and the remaining ones at higher temperatures (Table 1 and Figure S2). The decomposition process of the product [Cr_3_O(CH_3_CO_2_)_6_(Pri)(H_2_O)_2_] occurs in four stages, starting with the loss of water molecules (4.5%) up to approximately 97 °C. The loss of the more weakly bound, non-bridging acetate (see below) occurs in the region of 97–272 °C with an observed and calculated mass loss of 7.4%. The release of the flavonoid is observed in the region of 272–585 °C (with observed and calculated loss of 28.9% and 29.7%), followed by removal of acetate groups extending to and beyond 900 °C (Table 1 and Figure S2).

**Table 1:** Thermogravimetric data for the starting compounds [Cr_3_O(CH_3_CO_2_)_6_(H_2_O)_3_]Cl (Cr_3_O) and primuletin (HPri), and the product complex [Cr_3_O(CH_3_CO_2_)_6_(Pri)(H_2_O)_2_] (Cr_3_O-Pri).

Compound	Dehydration Process			Decomposition Process		
	Δ*T* (°C)	Δ*m*_exp_ (%)	Δ*m*_calc_ (%)	Δ*T* (°C)	Δ*m*_exp_ (%)	Δ*m*_calc_ (%)
Cr_3_O	25–158	14.5	14.4	158–600	39.5	38.3 (4Ac^−^)
HPri	–	–	–	190–232	100	100
Cr_3_O-Pri	25–97	4.5	4.6	97–272	7.4	7.4 (1Ac^−^)
				272–585	28.9	29.7 (Pri)
				585–860	22.2	22.1 (3Ac^−^)

Δ*T* = temperature range; Δ*m*_exp_ = experimental mass loss; Δ*m*_calc_ = calculated mass loss; Ac^−^ = CH_3_CO_2_^−^.

The ^1^H-NMR spectrum of primuletin was obtained in DMSO-*d*_6_ (Figure S3a). The four signals with δ 6.74 (1H, dd, *J* = 8.3 Hz; 1.0 Hz), δ 7.13 (1H, dd, *J* = 8.4 Hz; 1.0 Hz), δ 7.60 (1H, dd, *J* = 8.4 Hz and 8.3 Hz), and δ 8.03 (2H, dd, *J* = 8.1 Hz; 1.8 Hz) were assigned to H6, H8, H7 and H2'/H6', respectively. Additionally, two multiplets at δ 7.50 (2H) and δ 7.53 (1H) were assigned to H3'/H5' and H4’, respectively. The presence of two singlets at δ 7.01 (1H) and δ 12.6 (1H) were assigned to H3 and the hydrogen of the hydroxyl at C5. These assignments are in agreement with the literature [9,10]. The spectrum of the complex [Cr_3_O(CH_3_CO_2_)_6_(Pri)(H_2_O)_2_] in DMSO-*d*_6_ (Figure S3b) showed only a broad signal at δ 8.5 due to the high paramagnetism of the three Cr(III) ions, which promotes spin relaxation and absence of most signals. This typical paramagnetic behavior has been observed in other chromium complexes.

**Figure 2:**
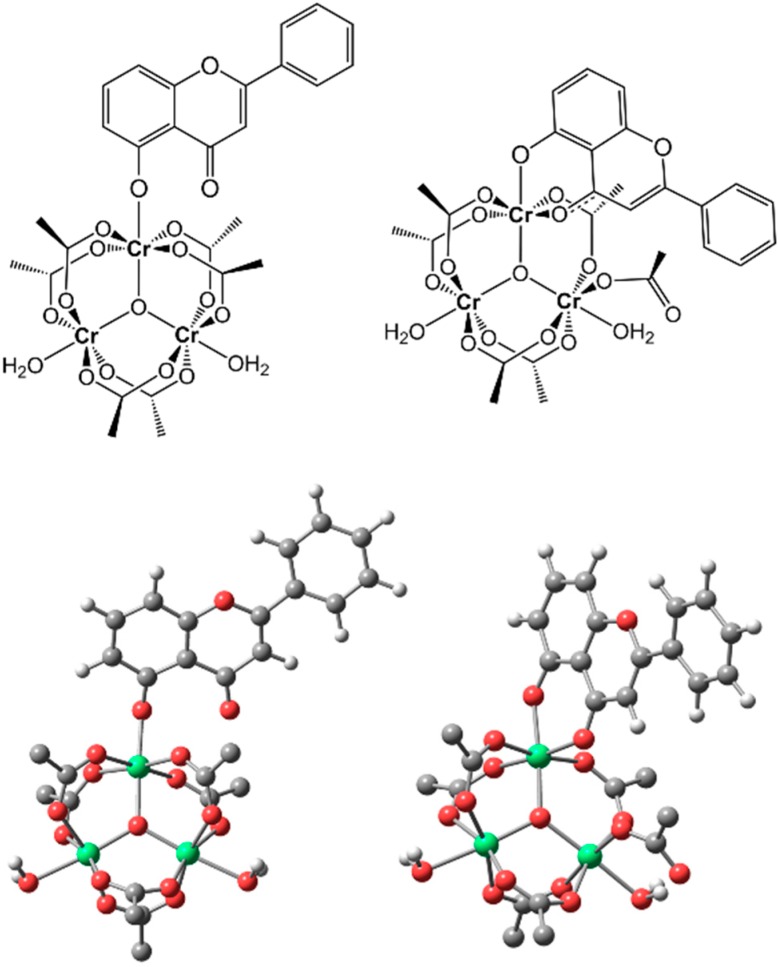
Drawing (**top**) and fully optimized density functional theory (DFT) structures (**bottom**) of the monodentate (**left**) and bidentate (**right**) isomers of the trinuclear Cr(III) complex with primuletin, formally [Cr_3_O(μ-CH_3_CO_2_)_6_(*O^5^*-Pri)(H_2_O)_2_] and [Cr_3_O(μ-CH_3_CO_2_)_5_(CH_3_CO_2_)(*O^5^,O^4^*-Pri)(H_2_O)_2_], respectively (the hydrogen atoms of acetate groups are omitted for clarity; color legend: Cr = green, O = red, and C = dark gray). Selected structural parameters are provided as Supplementary Material (Table S2).

Although primuletin is known to coordinate with metal ions as a bidentate ligand, trinuclear oxo-centered clusters of the type [M_3_O(CH_3_CO_2_)_6_(H_2_O)_3_]^+^ generally form complexes via substitution of the peripheral water ligands by monodentate species [11]. In order to elucidate which of these forms (*i.e.*, monodentate or bidentate) is favored in this specific flavonoid complex, the molecular structure of [Cr_3_O(CH_3_CO_2_)_6_(Pri)(H_2_O)_2_] was computationally studied by DFT calculations (see Computational Details). Full geometry optimizations at the B3LYP//6-31G*(C,H,O)/LANL2TZ(Cr) level were performed for both isomers shown in Figure 2: monodentate binding via the deprotonated hydroxyl (O5, ring *A*), and bidentate binding via hydroxyl (O5, ring *A*) and carbonyl (O4, ring *C*) groups. The geometry of the precursor [Cr_3_O(CH_3_CO_2_)_6_(H_2_O)_3_]^+^ was also optimized at the same level of theory for comparison with its reported crystal structure [12]; the calculated bond distances/angles are in excellent agreement with the experimental data (Table S1). Starting from this tris-aqua complex to form [Cr_3_O(CH_3_CO_2_)_6_(Pri)(H_2_O)_2_], the monodentate coordination of the flavonoid involves only the peripheral substitution of a water ligand, while the bidentate product involves additional displacement of an O atom of one of the six equatorially bridging acetates; that is, an acetate adjacent to the bidentate flavonoid loses its role as a bridge and becomes bound to a Cr(III) as a monodentate ligand (Figure 2). The DFT data clearly indicated that the isomer coordinated in a bidentate mode makes for the most stable structure. The energy difference favoring the bidentate isomer relative to the monodentate isomer is substantial: 15.68 kcal mol^−1^ (obtained at the B3LYP/6-311+G(2d,2p) level). This result is consistent with the mass spectra, which showed that the base peak has only five acetates. Moreover, the thermogravimetric analysis indicated the loss of one weakly bound acetate much before the other bridging acetate groups. A similar behavior was observed by Chaudhary and Van Horn [13].

**Figure 3:**
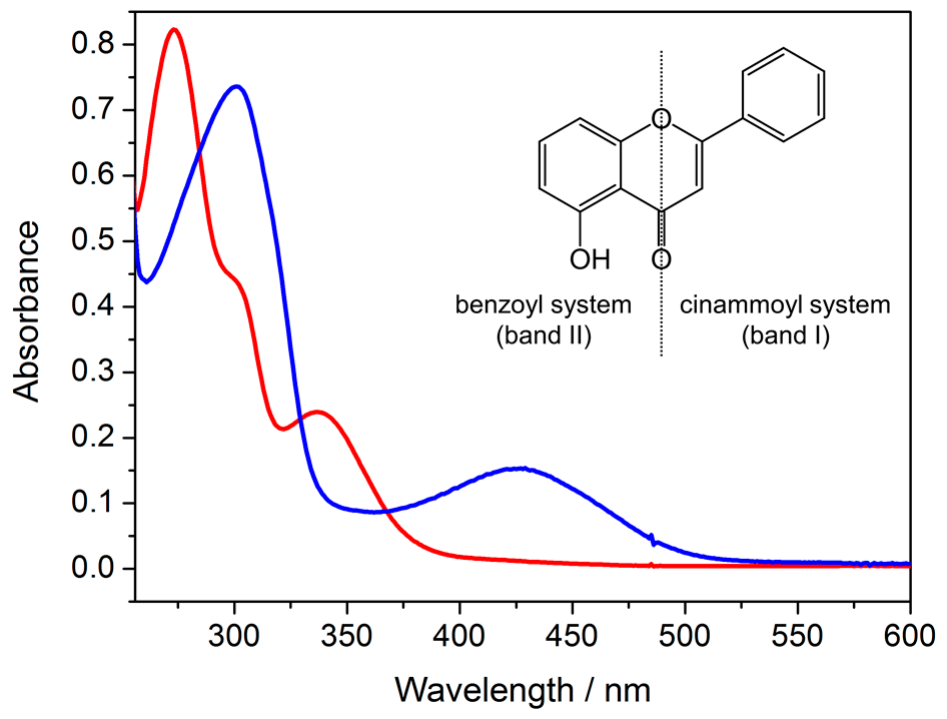
UV-Vis spectra of dimethylsulfoxide (DMSO) solutions of 43.1 μM primuletin (red line) and 18.8 μM [Cr_3_O(CH_3_CO_2_)_6_(Pri)(H_2_O)_2_] (blue line).

The UV-Vis absorption spectrum of free primuletin in DMSO (Figure 3 and Table 2) displays two defined bands at 273 nm and 337 nm and a shoulder around 300 nm. By comparison with the literature of flavonoids [10,14], the bands at 337 nm (band I) and 273 nm (band II) were assigned to π→π* electronic transitions originating at the flavonoid ring *B* (cinnamoyl system) and ring *A* (benzoyl system), respectively. For the complex [Cr_3_O(CH_3_CO_2_)_6_(Pri)(H_2_O)_2_], these bands are shifted to 426 nm and 301 nm, confirming the metal-flavonoid coordination. The spectra were obtained at different concentrations due the poor solubility of the complex.

**Table 2:** Absorption maxima (λ, nm) and molar absorptivities (ε, M^−1^·cm^−1^) of the main bands observed in the UV-Vis spectra of primuletin (HPri) and complex [Cr_3_O(CH_3_CO_2_)_6_(Pri)(H_2_O)_2_] (Cr_3_O-Pri) in DMSO, and starting complex [Cr_3_O(CH_3_CO_2_)_6_(H_2_O)_3_]^+^ (Cr_3_O) in ethanol.

Compound	Band II (π→π *)	Band I (π→π *)	Ligand Field (d→d)
HPri	273 (2.8 × 10^4^)	337 (8.0 × 10^3^)	–
Cr_3_O-Pri	301 (2.9 × 10^4^)	426 (8.1 × 10^3^)	– ^(a)^
Cr_3_O	–	–	442, 588 (<100)

^(a)^ Too weak; masked by the intense band I.

The IR spectra of primuletin and its complex are shown in Figure S4, and tentative assignments of the main peaks are collected in Table 3. The peak for the carbonyl stretching mode of primuletin appears at 1655 cm^−1^ [15]; following coordination to Cr(III), it is downshifted to 1626 cm^−1^ (Δν = 29 cm^−1^). This trend has been observed in the vibrational spectra of primuletin with other metals [16,17] and confirms the involvement of the carbonyl oxygen (O4) in the metal binding. The low-frequency vibrations in the region of 600–400 cm^−1^ are characteristic of the trinuclear cluster core, ν(Cr_3_O) and ν(CrO_4_) [11]. The two peaks at 627 and 525 cm^−1^ clearly indicate that the central Cr_3_O unit is preserved upon coordination to the flavonoid.

**Table 3:** FTIR data for free primuletin (HPri), precursor [Cr_3_O(CH_3_CO_2_)_6_(H_2_O)_3_] (Cr_3_O), and complex [Cr_3_O(CH_3_CO_2_)_6_(Pri)(H_2_O)_2_] (Cr_3_O-Pri).

Compound	Flavonoid			Acetate		Cr-O
	ν(C=O)	ν(C_2_=C_3_)	δ(C-OH)	ν_s_(COO)	ν_as_(COO)	
Cr_3_O	–	–	–	1450 vs	1611 vs	665 s 442 m
primuletin	1655 vs	1587 w	1319 vw	–	–	–
Cr_3_O-Pri	1626 vs	1576 w	–	1445 vs	1593 m	627 w
						525 w

Abbreviations: vs = very strong; s = strong; m = medium; w = weak; vw = very weak; ν = stretching; as = asymmetric; s = symmetric.

In the studies of antiradical properties, stock solutions of free and coordinated primuletin (0.1 mL, DMSO) were added to a 60 μM solution of DPPH (2,2-diphenyl-1-picrylhydrazyl) in methanol (3.9 mL). The reaction kinetics was followed by the change in absorbance at 515 nm during 45 min. To quantify the antiradical activity of the tested samples, the absorbance gradient (ΔAbs) was calculated by considering the absorbance of DPPH at time zero (*i.e.*, before addition of the samples) and then 45 min after mixing with the flavonoid samples [18]. From this procedure, the concentration of scavenged DPPH (DPPH_seq_) was calculated as [DPPH_seq_] = ΔAbs/(*b* × ε_515_), where ε_515_ is the molar absorptivity of DPPH at 515 nm (1.13 × 10^4^ M^−1^·cm^−1^) and *b* is the optical path length of the cuvette (1.0 cm). Thus, the number (*n*) of DPPH radicals sequestrated per molecule of the flavonoid sample (flav) was estimated as *n* = [DPPH_seq_]/[flav]. The flavonoid quercetin was used as standard antiradical in this work.

The obtained *n* values (Table 4) indicate that complexation led to an increase in the antiradical activity of the flavonoid, as *n* is higher for the complex compared to free primuletin. Further studies will be aimed at mechanistic details, although it is known that the antiradical activity of the flavonoid may occur via hydrogen atom transfer (HAT) and sequential proton loss electron transfer (SPLET) [19]. However, since primuletin becomes deprotonated upon coordination, SPLET is the only plausible mechanism in the complex [Cr_3_O(CH_3_CO_2_)_6_(Pri)(H_2_O)_2_]. This interpretation agrees with the observation that the complex [CrCl(Q)_2_(H_2_O)] (Q = quercetin) has a higher antiradical activity than that of free quercetin, from which it was concluded that Cr(III) is a better electron donor than the H atom [20].

**Table 4:** Antiradical activities of free and coordinated primuletin based on DPPH assays.

Compound	ΔAbs	[DPPH_seq_]	[flav]	*n*
HPri	0.017	1.50 × 10^−6^	1.01 × 10^−5^	0.155
Cr_3_O-Pri	0.020	1.77 × 10^−6^	2.50 × 10^−6^	0.734

ΔAbs = total change in absorbance for DPPH; [DPPH_seq_] = concentration of DPPH scavenged by the flavonoid sample; [flav] = flavonoid concentration; *n* = number of DPPH radicals scavenged per molecule of the flavonoid (free or coordinated).

## 3. Experimental Section

The starting compound [Cr_3_O(CH_3_CO_2_)_6_(H_2_O)_3_]Cl was synthesized as described in the literature [7]. All reagents and solvents were analytical grade. Elemental analyses (C, H) were performed using a Perkin Elmer CHN 2400. Molar conductance was measured for DMSO solutions (0.01–1.0 mM range) using a Digimed DM-32 conductivity meter. ESI mass spectra in positive mode were carried out with a Bruker Daltonics Esquire 3000 plus ESI-MS with dry temperature of 280 °C and capillary voltage of 4 kV; the sample was dispersed in acetonitrile and N_2_ was used as auxiliary gas. Thermogravimetric analyses were performed with a TA Instruments TGA 2950 Hi-Res thermogravimetric analyzer using 1–3 mg samples in a ceramic crucible, nitrogen flow at 50 mL·min^−1^, and heating rate of 10 °C·min^−1^. Infrared spectra from 4000 to 400 cm^−1^ were obtained with a Shimadzu FTIR 8400 instrument, using KBr pellets. Electronic absorption spectra in the region of 190–1100 nm were recorded on an Agilent 8453 UV-visible spectrophotometer. ^1^H-NMR spectra of complexes and free flavonoids in DMSO-*d*_6_ or CD_3_OD were collected on a Bruker DPX 300 spectrometer (^1^H/300 MHz).

### 3.1. Synthesis of [Cr_3_O(CH_3_CO_2_)_6_(Pri)(H_2_O)_2_]

The complex with primuletin (Pri) was prepared as reported in the literature [16]. A mixture containing 0.252 g (1.06 mmol) of primuletin, 0.040 g (1.01 mmol) of NaOH, 165 mL of ethanol, and 10 mL of water was heated under reflux for 15 h. Then, 0.221 g (0.33 mmol) of [Cr_3_O(CH_3_CO_2_)(H_2_O)_3_]Cl·3H_2_O in ethanol (5 mL) was added to the mixture under reflux. After 10 h of reaction, the solution was cooled to 5 °C and the obtained brown precipitate was filtered, washed with cold water and dried under vacuum. Yield: 26%. Anal. Calcd for C_27_H_31_O_18_Cr_3_ (MW = 799.5): C, 40.5; H, 3.91. Found: C, 40.2; H, 4.08.

### 3.2. Computational Details

Density functional theory (DFT) calculations were carried out using the Gaussian 09 program (revision D.01) [21]. Geometry optmizations without any constraints employed the hybrid B3LYP functional with the LANL2TZ relativistic effective core potentials and associated triple-zeta basis set for chromium and the 6-31G(d) basis set for all the other elements. In order to evaluate the quality of the results produced by this methodology, the structure of a reference system (the tris-aquo precursor [Cr_3_O(CH_3_CO_2_)_6_(H_2_O)_3_]^+^) was also computed and shown to be in excellent agreement with crystallographic data [12] (Table S1). Single-point energy calculations following geometry optimization were carried out using the triple-zeta 6-311+G(2d,2p) basis set; total energies for monodentate and bidentate isomers were −5536.514421 a.u. and −5536.539404 a.u., respectively. Since the complexes in this study have three Cr(III) centers with three unpaired electrons each (3*d*^3^), all calculations involved open-shell systems with spin multiplicity *M*_s_ = 10 (dectet).

## 4. Conclusions

In conclusion, the metal-ligand association between a trinuclear oxo-Cr(III) cluster and the flavonoid primuletin as ligand produced the complex [Cr_3_O(CH_3_CO_2_)_6_(Pri)(H_2_O)_2_]. The molecular structure of this product was elucidated through a combination of experimental and theoretical methods. The results showed that the Cr_3_O cluster core is retained upon complexation, and that the flavonoid binds to one of the metal centers in a bidentate mode via the 5-hydroxy/4-oxo groups. The studies involving the reaction between the free radical DPPH and the flavonoid (free and coordinated) indicated that complexation with the trinuclear cluster enhanced the antiradical activity of the flavonoid.

## Supplementary Materials

Supplementary materials can be accessed at: http://www.mdpi.com/1420-3049/20/04/6310/s1.
