# Supplementary material for: A Trinuclear Oxo-Chromium(III) Complex Containing the Natural Flavonoid Primuletin: Synthesis, Characterization, and Antiradical Properties
Source: Molecules. 2015 Apr 10;20(4):6310–8. doi: 10.3390/molecules20046310 (PMC6272679; doi:10.3390/molecules20046310)
Supplement: Supplementary file 1 [file molecules-20-06310-s001.pdf]

Supplementary Material

Table S1. Selected bond lengths (Å) and angles (deg). Comparison of the calculated (DFT) geometry of $[Cr_3O(CH_3CO_2)_6(H_2O)_3]^+$ and the experimental (X-ray) crystal structure of $[Cr_3O(CH_3CO_2)_6(H_2O)_3]Cl{\cdot}6H_2O$.

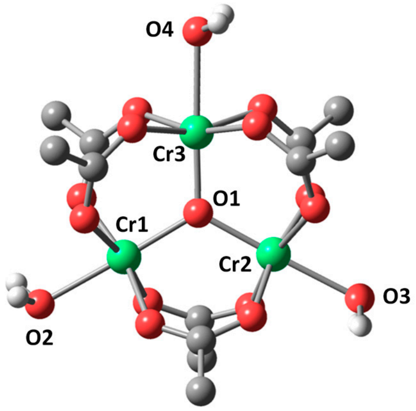

bond/Angle	Calculated [a]	Experimental [b]
O1-Cr1	1.90	1.90
O1-Cr2	1.90	1.90
O1-Cr3	1.89	1.88
avg Cr1-$O_{acetate}$	1.98	1.97
avg Cr2-$O_{acetate}$	1.98	1.96
avg Cr3-$O_{acetate}$	1.99	1.97
Cr1-O2	2.13	2.05
Cr2-O3	2.13	2.01
Cr3-O4	2.13	2.04
avg C-$O_{acetate}$	1.27	1.25
Cr1-O1-Cr2	119.8	119.9
Cr1-O1-Cr3	120.1	120.0
Cr2-O1-Cr3	120.1	120.1
O1-Cr1-O2	178.4	178.3
O1-Cr2-O3	178.3	177.9
O1-Cr3-O4	178.3	178.5
avg O1-Cr1-$O_{acetate}$	95.6	95.0
avg O1-Cr2-$O_{acetate}$	95.6	94.4
avg O1-Cr3-$O_{acetate}$	95.7	94.6
avg $O_{acetate}$-C-$O_{acetate}$	124.6	125.0
O1-$Pl_{Cr1Cr2Cr3}$	0.00[c]	0.02 [c]

[a] B3LYP//6-31G*(C,H,O)/LANL2TZ(Cr); see computational details; [b] X-ray crystal data from the literature for $[Cr_3O(CH_3CO_2)_6(H_2O)_3]Cl{\cdot}6H_2O$ (ref [12]); [c] Deviation from the mean Cr1-Cr2-Cr3 trigonal plan (Å).

Table S2. Selected bond lengths (Å) and angles (deg) from the calculated (DFT) geometry of [$Cr_3O(CH_3CO_2)_6$(Pri)$(H_2O)_2$]. [a]

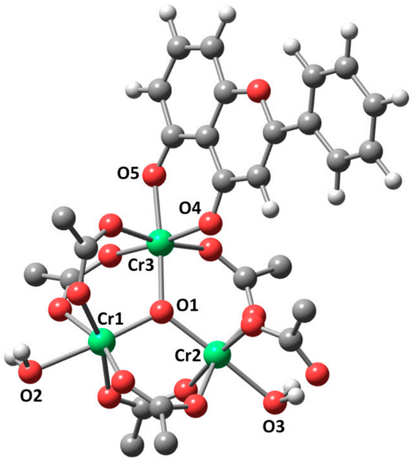

O1-Cr1	1.88
O1-Cr2	1.94
O1-Cr3	1.98
avg Cr1-$O_{acetate}$	1.99
avg Cr2-$O_{acetate}$	2.00
avg Cr3-$O_{acetate}$	2.01
Cr1-O2	2.18
Cr2-O3	2.09
Cr3-O4	1.97
Cr3-O5	1.96
O4-C4	1.27
O5-C5	1.29
Cr1-O1-Cr2	116.2
Cr1-O1-Cr3	117.5
Cr2-O1-Cr3	125.2
O1-Cr1-O2	176.2
O1-Cr2-O3	175.0
O1-Cr3-O4	93.7
O1-Cr3-O5	176.1
Cr3-O4-C4	128.8
Cr3-O5-C5	130.0
C4-C-C5	122.7
O1-$Pl_{Cr1Cr2Cr3}$	0.12[b]

[a] B3LYP//6-31G*(C,H,O)/LANL2TZ(Cr); see computational details; [b] Deviation from the mean Cr1-Cr2-Cr3 trigonal plan (Å).

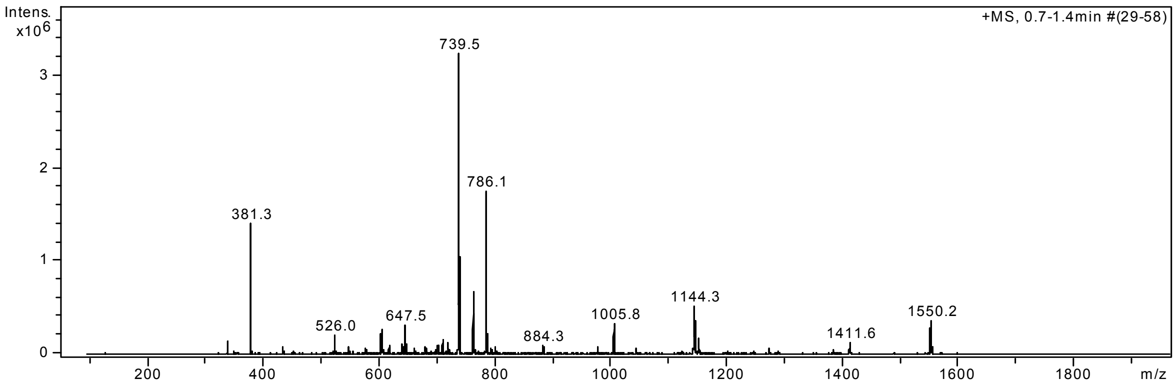

Figure S1. ESI MS of $[Cr_3O(CH_3CO_2)_6(Pri)(H_2O)_2]$ in acetonitrile.

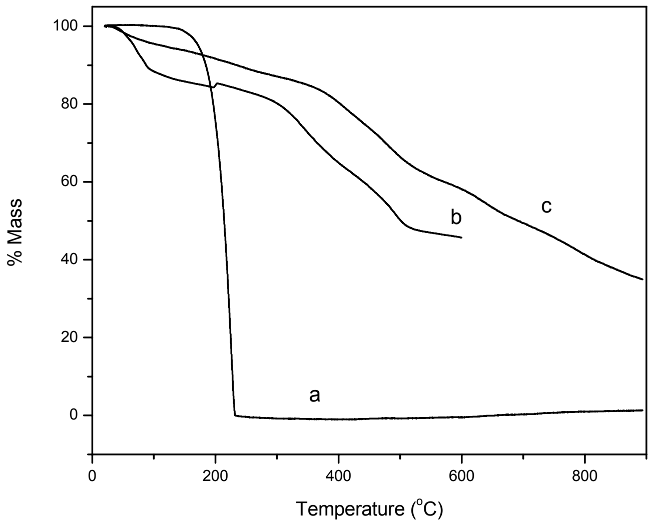

Figure S2. TG curve of (a) primuletin; (b) $[Cr_3O(CH_3CO_2)_6(H_2O)_3]Cl{\cdot}3H_2O$; and (c) $[Cr_3O(CH_3CO_2)_6(Pri)(H_2O)_2]$.

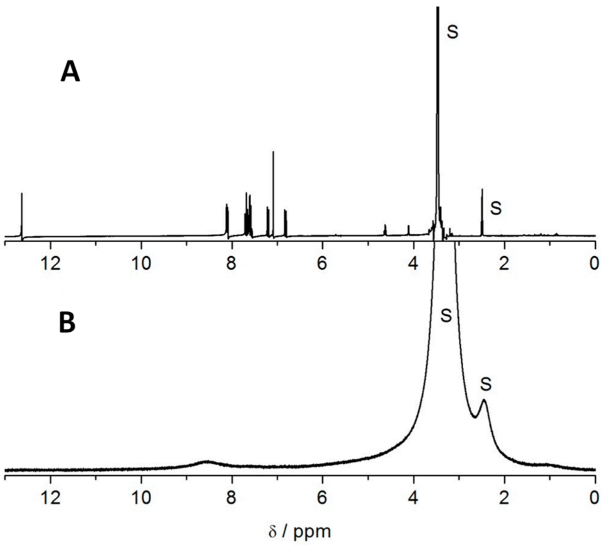

Figure S3. ^{1}H NMR spectra of (**A**) primuletin and (**B**) $[Cr_3O(CH_3CO_2)_6(Pri)(H_2O)_2]{\cdot}2H_2O$ in DMSO-d_6. Signals marked S represent the solvent.

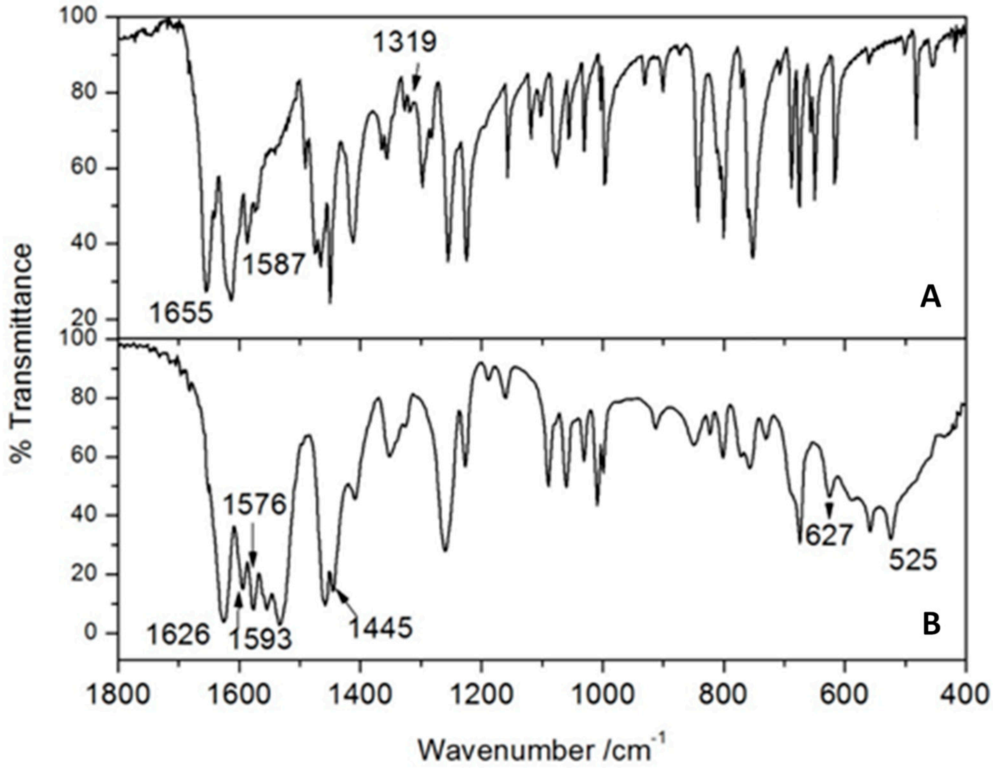

Figure S4. FTIR spectra of (**A**) primuletin and (**B**) complex [$Cr_3O(CH_3CO_2)_6(Pri)(H_2O)_2$] in KBr pellets.
